# MRI-guided focal or integrated boost high dose rate brachytherapy for recurrent prostate cancer

**DOI:** 10.3389/fonc.2022.971344

**Published:** 2022-08-26

**Authors:** Cynthia Ménard, Inmaculada Navarro-Domenech, Zhihu (Amy) Liu, Lisa Joseph, Maroie Barkati, Alejandro Berlin, Guila Delouya, Daniel Taussky, Marie-Claude Beauchemin, Benedicte Nicolas, Samuel Kadoury, Alexandra Rink, Srinivas Raman, Aravindhan Sundaramurthy, Robert Weersink, Dominic Beliveau-Nadeau, Joelle Helou, Peter Chung

**Affiliations:** ^1^ Radiation Oncology, Centre Hospitaliser de l’Université de Montréal (CHUM), Montreal, QC, Canada; ^2^ Princess Margaret Cancer Centre, University of Toronto, Toronto, ON, Canada; ^3^ Radiation Oncology, Polytechnique Montreal, Montreal, QC, Canada

**Keywords:** prostate cancer, brachytherapy, salvage, radiotherapy, magnetic resonance imaging

## Abstract

**Background and purpose:**

Locally recurrent prostate cancer after radiotherapy merits an effective salvage strategy that mitigates the risk of adverse events. We report outcomes of a cohort enrolled across two institutions investigating MRI-guided tumor-targeted salvage high dose rate brachytherapy (HDR-BT).

**Materials and methods:**

Analysis of a prospective cohort of 88 patients treated across two institutions with MRI-guided salvage HDR-BT to visible local recurrence after radiotherapy (RT). Tumor target dose ranged from 22-26 Gy, using either an integrated boost (ibBT) or focal technique (fBT), delivered in two implants over a median of 7 days. Outcome metrics included cancer control and toxicity (CTCAE). Quality of life (QoL-EPIC) was analyzed in a subset.

**Results:**

At a median follow-up of 35 months (6 -134), 3 and 5-year failure-free survival (FFS) outcomes were 67% and 49%, respectively. At 5 years, fBT was associated with a 17% cumulative incidence of local failure (LF) outside the GTV (vs. 7.8% ibBT, p=0.14), while LF within the GTV occurred in 13% (vs. 16% ibBT, p=0.81). Predictors of LF outside fBT volumes included pre-salvage PSA>7 ng/mL (p=0.03) and interval since RT less than 5 years (p=0.04). No attributable grade 3 events occurred, and ibBT was associated with a higher rate of grade 2 toxicity (p<0.001), and trend towards a larger reduction in QoL sexual domain score (p=0.07), compared to fBT.

**Conclusion:**

A tumor-targeted HDR-BT salvage approach achieved favorable cancer control outcomes. While a fBT was associated with less toxicity, it may be best suited to a subgroup with lower PSA at later recurrence. Tumor targeted dose escalation may be warranted.

## Introduction

Despite improvements in prostate cancer treatment delivery allowing dose escalation of radiotherapy (RT), local persistence or recurrence of disease remains prevalent in a substantial subset of patients (1). Local disease after RT is a risk factor for subsequent metastatic progression and prostate cancer-specific mortality (2–4), and is a cause of morbidity including hematuria, obstructive uropathy, and chronic pain (5).

Following RT for localized prostate cancer, biochemical failure (BF) may be attributed to local recurrence and confirmed through MRI and biopsy (6), if staging investigations fail to identify distant disease. Indicators of local-only failure include favorable clinical pre-treatment characteristics and a slow PSA doubling time (7). Patients with these features have the potential to benefit from local salvage treatment (8). Moreover, the use of salvage brachytherapy has been associated with low rates of severe toxicity (9–11).

A recent meta-analysis including 150 publications supports the role of local salvage interventions, with 50-60% 5-year failure-free survival outcomes achieved, regardless of the salvage modality used (12), including salvage prostatectomy. However, rates of grade >3 toxicity may be significant in surgical approach, ranging from 4-23%, whereas radiotherapeutic salvage series generally fair more favorably.

More recently, ‘focal’ or partial gland salvage has been increasingly studied in the hopes of further reducing the toxicity of salvage therapy (13–15). Given the high performance of MRI in identifying sites of prostate recurrence (16), there is a strong rationale for MRI-guided and tumor-targeted approach to improve the therapeutic ratio. The aim of this study was to report outcomes achieved with this strategy, and to examine the performance of focal and integrated boost salvage high dose rate brachytherapy (HDR-BT).

## Materials and methods

Patients were enrolled prospectively on REB-approved registry trials at two institutions and completed MRI-guided and tumor-targeted HDR salvage brachytherapy between 2009 and 2020 (NCT00913939; NCT03378856).

### Patient selection

Eligibility included biochemical failure (PSA nadir + 2 ng/mL) (17) with MRI-visible and histologically confirmed local recurrence occurring more than 18 months after definitive RT and/or BT. Patients with IPSS ≤ 18, ECOG 0 or 1, and no radiological evidence of distant metastases were included. All patients completed a staging exam prior to salvage HDR-BT including bone scan, abdomen-pelvis CT (+/- thorax) and/or 18F-DCFPyL PSMA-PET. Patients with inflammatory bowel disease and/or contraindications to MRI or general anesthesia were excluded.

### Salvage HDR-BT

Brachytherapy planning consisted of a multiparametric MRI (mpMRI) compliant with PI-RADS acquisition guidelines (18), with/without 18F-DCFPyL PSMA-PET, where the GTV was delineated to represent nodular regions of restricted diffusion, early contrast enhancement, and radiotracer uptake corresponding to pathologically confirmed sites. Brachytherapy was then performed and planned under MRI-only or MRI-TRUS guidance (depending on the institution) using either, an integrated boost (ibBT) or focal (fBT) technique. The tumor CTV included the GTV, and 5 mm margin restricted up to 2 mm beyond the prostate and excluding urethra. A 2 mm superior/inferior PTV margin was applied to the tumor CTV to account for volume averaging and registration uncertainties. A whole prostate CTV (CTVwp) was routinely included in the early part of the study period while gaining experience with tumor targeting. In the latter cases, CTVwp was included at the discretion of the treating physician when there was multifocal disease or the presence of non-visible biopsy-proven microscopic disease at intra-prostatic sites distant to the GTV. Organs at risk (OARs), including the urethra and rectum, were delineated as a solid organ.

GTV dose ranged from 22-26 Gy, while CTVwp dose ranged from 16-22 Gy (ibBT group only) in two brachytherapy implants delivered 5-14 days apart. Treatment plans were generated to achieve a GTV V100 > 99%, and PTV V100>95%, respecting normal tissue dose constraints. Dose planning objectives for OARs were rectum D0.5cc < 10Gy, urethra D0.5cc < 13 Gy or urethra DMax <16 Gy per implant.

**Figure 1:**
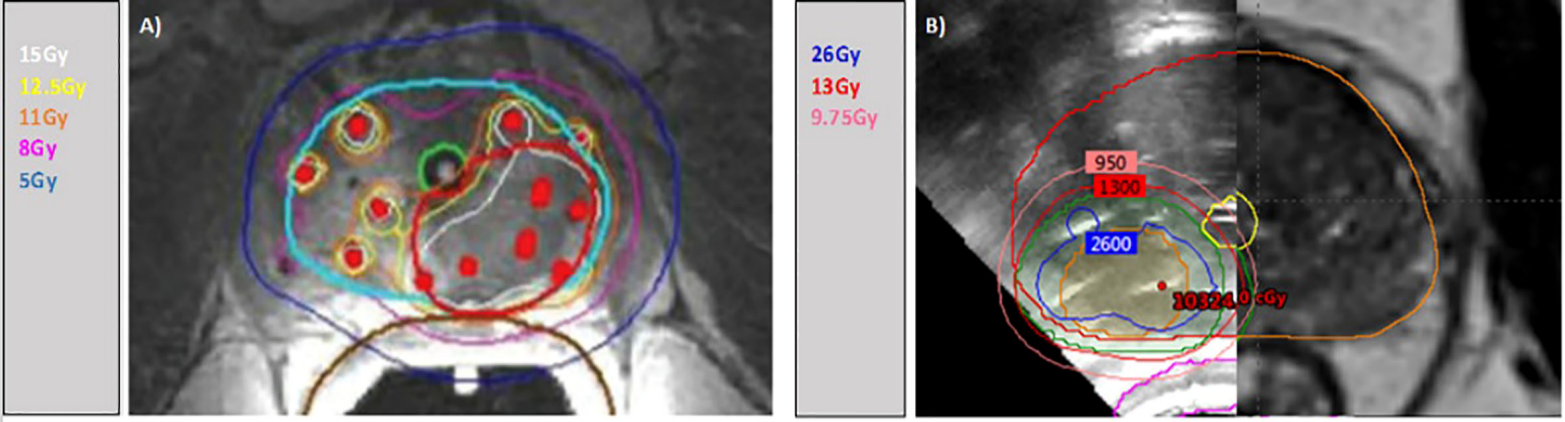
Isodose distributions for case examples. **(A)** ibBT with MRI-only guidance. Contoured in light blue the CTVwp, in red the PTV (GTV). Isodose lines: white 15Gy (135%), yellow 12.5Gy (114%), orange 11Gy (100%), pink 8Gy (75%) and dark blue 5Gy (50%). **(B)** fBT with TRUS/MRI guidance. Contoured in orange the GTV and green the PTV (GTV). Isodose lines: blue 26Gy (200%), red 13Gy (100%), pink 9.75Gy (75%).

Adjuvant short-course (4-6 months) of androgen deprivation therapy (ADT) was permitted at the discretion of the treating physician based on the presence of Gleason Grade Group 4 or 5 disease at the time of salvage. A case example of each technique is depicted in **Figure 1**.

### Follow-up

Genitourinary and gastrointestinal toxicity was graded using CTCAE v4. The quality of life (QoL) for urinary, bowel and sexual domains was measured using EPIC at baseline, and at 1, 3, 6, 12, 24, 36 and 60 months post salvage in a subset. Subsequent biochemical failure events were investigated with conventional staging investigations, prostate MRI, and biopsy when persistent/recurrent disease was visualized on MRI.

### Statistical analysis

Summary statistics were used to describe patient, disease and treatment characteristics. Wilcoxon’s rank sum test and Fisher’s exact test were used to compare continuous and categorical variables respectively. Failure-free survival (FFS) was defined as the time from HDR brachytherapy salvage to the first occurrence of biochemical failure (BF), local failure (LF), regional failure (RF), distant metastasis (DM), or death from any cause. BF was defined as nadir + 2ng/ml from the post-salvage PSA nadir. LF was defined as any intraprostatic recurrence (including seminal vesicles) and analyzed as either in-field (at least partly within the salvage PTV-GTV), or out-of-field. RF was defined as pelvic lymph node metastases. Kaplan-Meier (KM) curves and logrank test were used to compare FFS in each cohort. Cumulative incidence function was used for LF, and next-line treatment considering death as a competing risk. Gray’s test was used to compare cumulative incidence in fBT and ibBT.

Univariate and multivariable Cox proportional hazards model was fitted to assess potential prognostic factors for failure. Mean change score from baseline and their 95% confidence intervals (CI) were presented over time for urinary, bowel and sexual scores. Multivariable linear mixed effects model was fitted to compare the change score in sexual function over time in ibBT and fBT cohorts, with baseline score (continuous variable) and time (categorical variable) as fixed effects and intercept as a random effect to allow intercepts to vary between individuals. All tests were two sided, and a p-value < 0.05 was considered as statistically significant.

## Results

Of 90 patients enrolled, two patients did not complete treatment due to anesthetic events and were excluded from analysis, leaving 88 patients with a median follow-up of 35 months (range 6-134). Groups were balanced in terms of risk factors at initial diagnosis (Gleason Grade Group (GG), T-stage, PSA, NCCN risk group), and prior RT dose. Most of the patients underwent definitive EBRT (72%) and the mean EQD2 dose was 78Gy (range 62,115). Patients with prior LDR brachytherapy (25%) all received fBT. Median follow-up was longer in the ibBT cohort, while a higher (GG) and more advanced local disease at salvage was found in the fBT cohort. Thirty-two patients (44%) from this group, completed a PSMA PET as part of staging. Twenty-six (30%) of patients received adjuvant short-course ADT. Patient and treatment characteristics are depicted in **Table 1**.

**Table 1:** Patient and treatment characteristics (cohort differences in bold).

Variable	Cohort (n=88)	ibBT (n=15)	fBT (n=73)	p-value
Initial Gleason Grade Group (GG) (5)				0.76
1-3	79 (91%)	15 (100%)	44 (89%)	
4-5	8 (9%)	0	8 (11%)	
Initial “T-stage”				0.56
T1-T2	81 (92%)	14 (93%)	67 (92%)	
T3-T4	6 (7%)	1 (7%)	5 (7%)	
Prior definitive radiotherapy (RT) modality				0.12
External Beam RT (EBRT)	63 (72%)	14 (93%)	49 (67%)	
Low Dose Rate brachytherapy (LDR-BT)	18 (20%)	0	18 (25%)	
EBRT + BT boost	7 (8%)	1 (7%)	6 (8%)	
Age at salvage (median, range)	71 (56, 85)	69 (62, 75)	71 (56,85)	0.06
Median RT interval (year, range)	7 (3,17)	6 (3,12)	7 (3,17)	0.09
Recurrence Gleason GG (%)				**0.03**
1-3	54 (65%)	13 (93%)	41 (59%)	
4-5	29 (35%)	1 (7%)	**28 (41%)**	
Recurrence MRI “T-stage” (%)				**0.02**
T2	68 (77%)	15 (100%)	53 (73%)	
T3-4	20 (23%)	0	**20 (27%)**	
Median pre-salvage PSA (range)	4.5 (1.2, 24)	5.4 (2.1, 14.5)	4.2 (1.2, 24)	0.67
Median PSA doubling time (DT) (months, range)	15 (4, 55)	12 (5, 33)	16 (4, 55)	0.46
Number of target lesions				0.34
1 target lesion	81 (92%)	13 (87%)	68 (93%)	
2 target lesions	7 (8%)	2 (13%)	5 (7%)	
Adjuvant short-course androgen deprivation therapy (ADT) (%)	26 (30%)	2 (13%)	24 (33%)	0.21
Median follow-up (months, range)	35 (6,134)	**98 (17, 134)**	29 (6, 86)	**<0.001**

**Figure 2:**
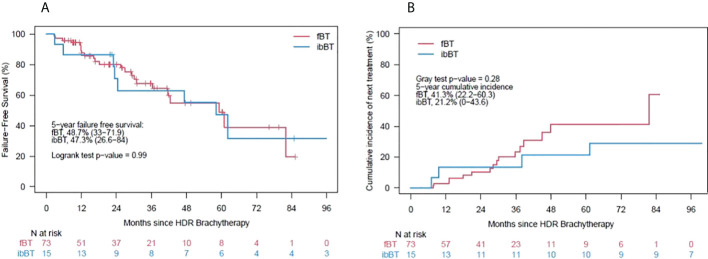
**(A)** – Kaplan-Meier curves for failure-free survival, **(B)** – cumulative incidence of next-line therapy.

After salvage BT, the nadir PSA decreased to a median of 0.3 ng/mL (range 0-11.2) in those patients who did not receive adjuvant ADT. The 3 and 5-year FFS was 67% (95% CI 56 - 80) and 49% (95% CI 36 - 67), respectively. There was no difference in FFS outcomes between the fBT and ibBT cohorts, **Figure 2**. The cumulative incidence of distant metastasis was 4% (0-8) and 14% (2-25) at 3 and 5 years, with no difference between fBT and ibBT cohorts (p=0.24). At the time of analysis, two patients had died of metastatic prostate cancer.

There was a higher incidence of next-line therapeutic interventions in the fBT cohort at 5 years, but this was not statistically significant (41 vs. 21%, p=0.28), **Figure 2**. Next-line therapy was initiated in 23 (26%) patients [16 (22%) patients in the fBT vs. seven (47%) in ibBT group]. ADT alone, most commonly LHRH analogue injection, was used in 17 of those patients, and combined with androgen receptor-axis-targeted therapies (ARAT) in three patients. Of remaining patients, further local therapy such as high-intensity focused ultrasound (HIFU) or radiation treatment was used.

**Figure 3:**
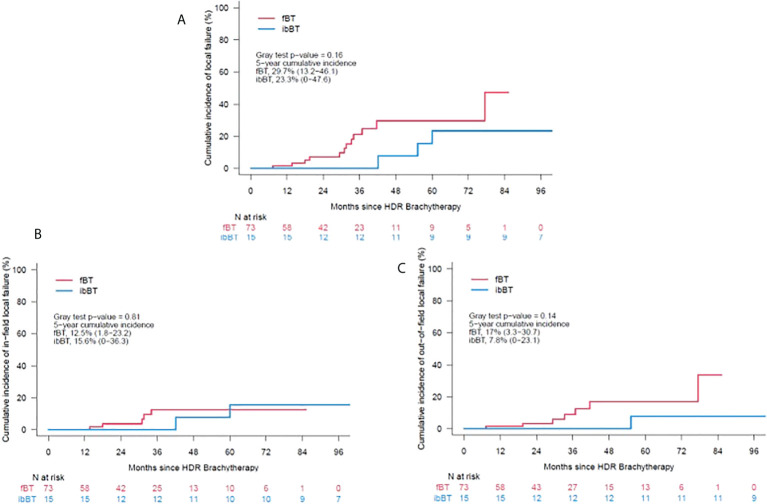
**(A)** – Cumulative incidence of local failure, **(B)** – Cumulative incidence of in-field failure, **(C)** –Cumulative incidence of out-of-field failure.

At 3 years, fBT was associated with a 21% cumulative incidence of local failure (LF) and none in the ibBT cohort (p=0.07), but this difference was not sustained at 5 years (30% vs. 23%, p=0.16). In further analysis, the rate of in-field failure at 5 years was similar between fBT and ibBT techniques (13% vs.16% respectively, p=0.81). However, the rate of out-of-field failures diverged, trending to a higher incidence at 5 years for the fBT vs. ibBT technique (17% vs. 8%, p=0.14), **Figure 3**.

Univariate analysis was performed to examine factors predictive of outcome. A higher PSA at the time of salvage, and a shorter time interval since initial radiotherapy were predictive of local failure. Gleason grade group (GG) 4-5 at recurrence was also predictive of out-of-field local failure in the fBT cohort. In contrast, high-risk characteristics at initial diagnosis (GG4-5 or NCCN high risk) predicted for regional and distant metastasis after local salvage (**Table 2**). The use of ADT was not a statistically significant predictive factor.

**Table 2:** Univariate analysis of predictive factors of outcomes.

Outcome	Covariate	HR (95%CI)	p-value
**Any failure**	PSA at recurrence (ng/mL)	1.19 (1.11, 1.29)	<0.001
	Radiation Treatments (RT) interval (year)	0.86 (0.75, 0.98)	0.02
**Local failure**	PSA at recurrence (ng/mL)	1.15 (1.03, 1.28)	0.01
	RT interval (year)	0.76 (0.6, 0.95)	0.02
**Out-of-field local failure(fBT cohort)**	Gleason Grade Group (GG) 4-5 at recurrence (reference: GG 1-3)	4.59 (0.9, 23.58)	0.07
	PSA at recurrence (ng/mL)	1.16 (1.00, 1.33)	0.04
	RT interval (year)	0.70 (0.48,1.01)	0.06
**Regional or distant metastasis**	Gleason GG 4-5 at diagnosis (reference: GG 1-3)	2.85 (0.90,9.08)	0.08
	High Risk (NCCN) at diagnosis (reference: low risk)	9.61 (1.13, 81.37)	0.04

In multivariable analysis, for FFS both pre-salvage PSA and RT interval maintained significance; p<0.001 and 0.02, respectively. Finally, the following thresholds reached statistical significance for predicting out-of-field local failures, beyond the PTV, in the fBT cohort: PSA>7ng/ml (p=0.03), and RT interval ≤5 years (p=0.04).

There was no grade 3 or higher toxicity events attributable to salvage BT. One patient developed late grade 3 urinary retention attributable to tumor progression. Fewer grade 2 GU and GI toxicity events occurred in patients treated with fBT compared with ibBT (p<0.001) (**Table 3**).

**Table 3:** Toxicity events attributable to salvage brachytherapy (CTCAE grade).

Domain and grade	Cohort (n=88)	fBT (n=73)	ibBT (n=15)	p-value
**Genitourinary**				**<0.001**
0	39 (44%)	38 (52%)	1 (7%)	
1	29 (33%)	24 (33%)	5 (33%)	
2	20 (23%)	**11 (15%)**	**9 (60%)**	
**Gastrointestinal**				**<0.001**
0	77 (88%)	69 (95%)	8 (53%)	
1	8 (9%)	4 (5%)	4 (27%)	
2	3 (3%)	**0 (0%)**	**3 (20%)**	

Bold highlighting differences.

**Figure 4:**
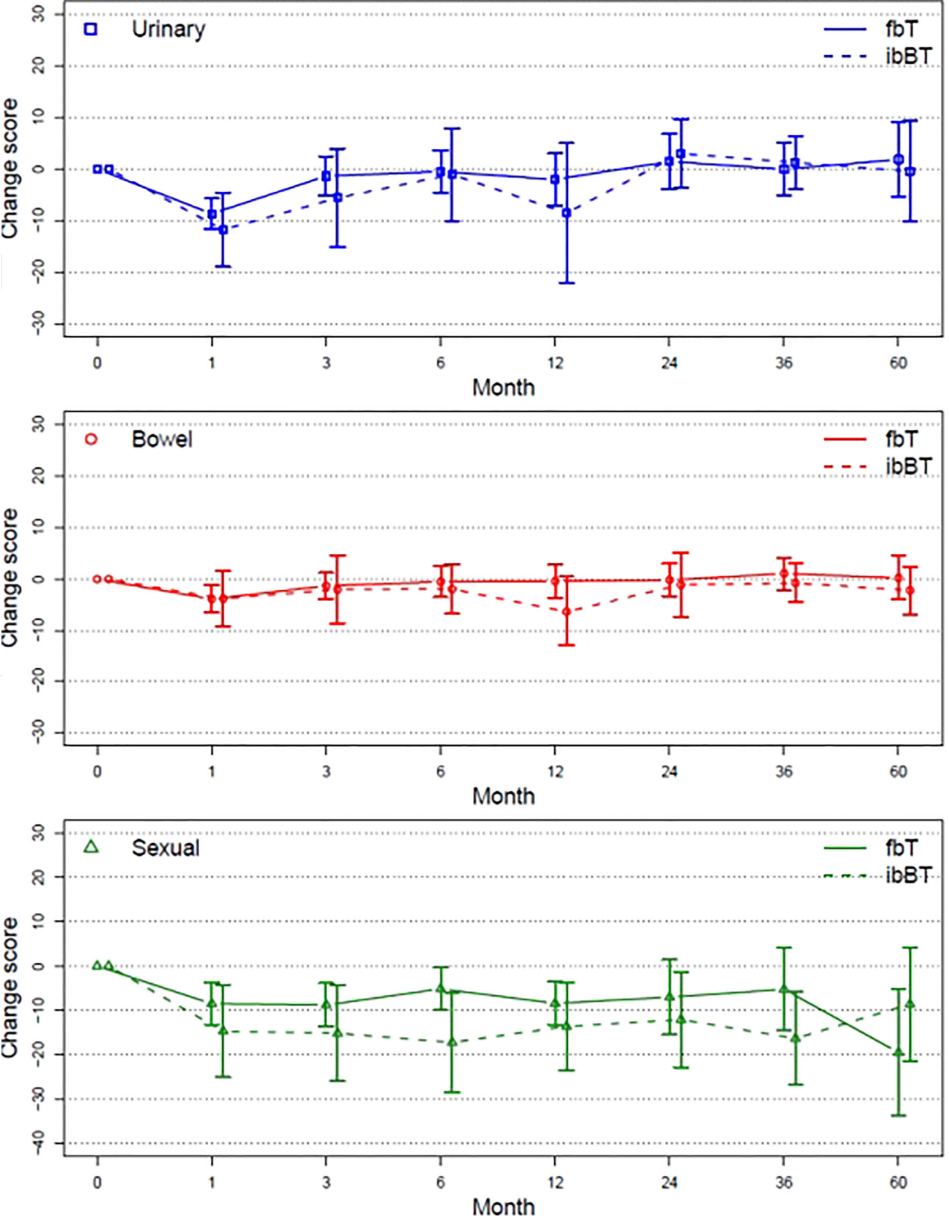
Mean change score from baseline (95% CI) plotted over time for urinary, bowel and sexual QoL.

The impact of salvage BT on QoL was analyzed in subset of 50 patients of whom 13 (26%) underwent ibBT and 37 (74%) fBT. Urinary and bowel QoL returned to baseline 3 months after salvage BT, while sexual function decline persisted (**Figure 4**). ibBT was associated with a trend to a larger reduction in QoL sexual domain score from baseline compared with fBT (p=0.07).

## Discussion

We report a large series of MRI-guided, tumor-targeted HDR brachytherapy for locally recurrent prostate cancer. Overall, we found 3 and 5-year FFS outcomes comparable to those achieved with whole-gland ablative modalities (12), where cancer control was achieved with minimal toxicity and no grade ≥3 events attributed to salvage brachytherapy. We also demonstrated that focal HDR implants were better tolerated than whole-gland dose-painted implants.

Some of the largest series for whole gland salvage brachytherapy, including RTOG-0526, reported G3 toxicity rates between 14-27% (19, 20) with LDR and 21% (20) with HDR brachytherapy salvage. In other series of HDR focal salvage, rates of late Grade 3 toxicity were low ranging from 3% to 10% (13, 15). Systematic reviews of SBRT salvage (12, 21), found comparably low rates of severe toxicity (2-4%). Similarly, partial prostate treatment also faired more favorably than whole-gland treatment with reported 2-year FFS of 62% (47–74). In our series the 2-year FFS for fBT was 80.1% (95% CI 70.6%-91.0%) but as expected by 5 years rates were similar to the published literature. We found that this evident gain in toxicity reduction of local salvage through tumor-targeting may be partially offset with a compromise in subsequent out-of-field intra-prostatic local control.

As both mpMRI and PET imaging performance likely improves selection of appropriate patients in the salvage setting after radiotherapy (22–24), we may expect local control to improve in parallel. Many recent advances, including the publication of PI-RR MRI guidelines (16) and mounting availability of PSMA-PET will enhance the detection and hence the image-guidance for focal salvage interventions. Selection of a subset of patients at particular risk of out-of-field intra-prostatic failures is challenging, but PSA > 7ng/mL, time from initial radiotherapy less than 5 years, and Gleason GG 4 or 5 at failure appeared to be important factors in our cohort. We hypothesize that such patients may benefit from either the application of larger PTV margins, or a dose-painted approach to address regions at higher risk of bearing microscopic disease undetectable by imaging.

Approximately 15% of patients had in-field failure despite a delivered EQD2 of 110 Gy (2 fractions, alpha/beta = 1.4). It is entirely plausible that patients who recur after radiotherapy indeed have more radioresistant disease and may require higher doses in order to achieve durable local control. Given the very low rates of toxicity observed, we feel there is merit to further dose escalate the GTV.

It is noteworthy that regional and distant metastatic failures after local salvage were predominantly predicted by disease characteristics at first diagnosis (baseline Gleason GG and NCCN risk group), rather than disease characteristics at the time of failure. This indicates that staging with PSMA-PET may be most warranted in patients initially presenting with high-risk disease before offering local salvage interventions. Furthermore, MR-assessed T3 and GG 4 disease at recurrence may not confer the same risk of distant failure as at original diagnosis and thus may not necessarily preclude the use of local salvage in such patients. It remains that local disease after RT is a major problem for patient leading to subsequent metastatic progression and is a cause of morbidity including hematuria, obstructive uropathy, and chronic pain (25). A limitation of our study is the lack of genomic-prognostic analysis, which stands to further define which subset of patients are best suited for focal or whole-gland salvage interventions (26).

However, the prospective nature of our approach aligned across two institutions in a well-defined population and with meaningful follow-up is a key strength of our study. While the comparison of two clearly imbalanced sub-cohorts (fBT vs. ibBT) in *post-hoc* analysis presents important limitations, such comparisons can help us generate hypotheses on future directions in order to further improve patient outcomes.

Although this study includes a reasonably large number of patients, this is a selected population that were suitable for this type of approach. Many patients that have local failure after previous radiotherapy opt for more conservative management strategies such as delayed ADT. In our cohort, local salvage delayed the use of long-term HT with 59% (fBT) and 79% (ibBT) of patients free of next-line HT at 5 years. It should be noted that there is limited long term follow-up in our study such that estimates of long term cancer outcomes may be less robust, however, it is reassuring that such outcomes are consistent with the available literature in this setting.

For future research, we believe it is necessary for broad collaboration to address a number of outstanding questions in this space, through either randomized cooperative group trials, or alternatively large-scale registries. Key questions remain, including the role of ADT and the choice of SBRT vs. BT. Efforts to achieve consensus on best practice given available data are also needed.

## Conclusions

A tumor-targeted HDR-BT salvage approach achieves favorable patient outcomes. While a focal salvage technique limits toxicity, it may be best suited to a subgroup with lower PSA and Gleason at late recurrence. Tumor dose escalation beyond EQD2 110 Gy may also be warranted to improve local control.

## Data availability statement

The raw data supporting the conclusions of this article will be made available by the authors, without undue reservation.

## Ethics statement

The studies involving human participants were reviewed and approved by Research Ethics Board (REB). The patients/participants provided their written informed consent to participate in this study.

## Author contributions

These authors have contributed equally to this work and share first authorship. All authors contributed to the article and approved the submitted version.

## Funding

Grant funding provided in part by DOD PCRP, NIH R21CA121586-01A2, CARO-ACURA, CRCHUM, and Varian Medical Systems through the PERa Network (Partnership for the Evaluation of Innovation in Radiotherapy).

## Acknowledgments

Authors would like to acknowledge Drs. Tamim Niazi, Gerard Morton, Michael Milosevic, Charles Catton, Padraig Warde, Boris Bahoric, and Andrew Bayley for referrals in consideration of research in salvage HDR brachytherapy; Drs. Masoom Haider, Sangeet Ghai, Daniel Juneau, and Damien Olivier for support in imaging; Dr. Trish Pulvirenti, Ms. Anna Simeonov, Ms. Bernadeth Lao, Ms. Mom Phat, and Dr. Gerald O’Leary for support in clinical research and patient management.

## Conflict of interest

The authors declare that the research was conducted in the absence of any commercial or financial relationships that could be construed as a potential conflict of interest.

## Publisher’s note

All claims expressed in this article are solely those of the authors and do not necessarily represent those of their affiliated organizations, or those of the publisher, the editors and the reviewers. Any product that may be evaluated in this article, or claim that may be made by its manufacturer, is not guaranteed or endorsed by the publisher.
