# Corrigendum: MRI-guided focal or integrated boost high dose rate brachytherapy for recurrent prostate cancer

**DOI:** 10.3389/fonc.2022.1084708

**Published:** 2022-11-17

**Authors:** Cynthia Ménard, Inmaculada Navarro-Domenech, Zhihu (Amy) Liu, Lisa Joseph, Maroie Barkati, Alejandro Berlin, Guila Delouya, Daniel Taussky, Marie-Claude Beauchemin, Benedicte Nicolas, Samuel Kadoury, Alexandra Rink, Srinivas Raman, Aravindhan Sundaramurthy, Robert Weersink, Dominic Beliveau-Nadeau, Joelle Helou, Peter Chung

**Affiliations:** ^1^ Radiation Oncology, Centre Hospitaliser de l’Université de Montréal (CHUM), Montreal, QC, Canada; ^2^ Princess Margaret Cancer Centre, University of Toronto, Toronto, ON, Canada; ^3^ Radiation Oncology, Polytechnique Montreal, Montreal, QC, Canada

**Keywords:** prostate cancer, brachytherapy, salvage, radiotherapy, magnetic resonance imaging

In the published article, there was an error in the author list. In the published manuscript the author list appears erroneously: Cynthia Ménard¹*†, Inmaculada Navarro-Domenech²†, Zhihu (Amy) Liu², Lisa Joseph², Maroie Barkati¹, Alejandro Berlin², Guila Delouya¹, Daniel Taussky¹, Marie-Claude Beauchemin¹, Benedicte Nicolas¹, Samuel Kadoury³, Alexandra Rink², Srinivas Raman², Aravindhan Sundaramurthy², Robert Weersink², Dominic Beliveau-Nadeau¹, Joelle Helou², and Peter Chung² on behalf of NIKE.

The corrected author list is as appears below.

Cynthia Ménard¹*†, Inmaculada Navarro-Domenech²†, Zhihu (Amy) Liu², Lisa Joseph², Maroie Barkati¹, Alejandro Berlin², Guila Delouya¹, Daniel Taussky¹, Marie-Claude Beauchemin¹, Benedicte Nicolas¹, Samuel Kadoury³, Alexandra Rink², Srinivas Raman², Aravindhan Sundaramurthy², Robert Weersink², Dominic Beliveau-Nadeau¹, Joelle Helou², and Peter Chung²

The authors apologize for this error and state that this does not change the scientific conclusions of the article in any way. The original article has been updated.

## Publisher’s note

All claims expressed in this article are solely those of the authors and do not necessarily represent those of their affiliated organizations, or those of the publisher, the editors and the reviewers. Any product that may be evaluated in this article, or claim that may be made by its manufacturer, is not guaranteed or endorsed by the publisher.

